# Network meta-analysis of non-pharmacological interventions for cognitive impairment after an ischemic stroke

**DOI:** 10.3389/fneur.2024.1327065

**Published:** 2024-06-04

**Authors:** Guangming Yang, Liyun Guo, Yuan Zhang, Shan Li

**Affiliations:** Department of Rehabilitation Medicine, Heping Hospital Affiliated to Changzhi Medical College, Changzhi, China

**Keywords:** ischemic stroke, cognitive impairment, network meta-analysis, randomized controlled trials, non-pharmacological interventions

## Abstract

**Objective:**

This study aims to evaluate the effectiveness of non-pharmacological interventions in improving cognitive function in patients with ischemic stroke through network meta-analysis.

**Methods:**

We searched databases including the Cochrane Library, PubMed, EmBase, and Web of Science for randomized controlled trials (RCTs) on non-pharmacological treatments to improve cognitive impairment following ischemic stroke. The publication date was up to 15 March 2023. Due to the insufficiency of included studies, supplementary searches for high-quality Chinese literature were performed in databases such as CNKI, WanFang Data, and VIP Chinese Science Journals Database. Two reviewers independently went through the literature, extracted data, and assessed the risk of bias in the included studies using the risk of bias assessment tool recommended by the Cochrane Handbook for Systematic Reviews of Interventions 5.1.0. By utilizing R 4.2.3 RStudio software and the GeMTC package, a Bayesian network meta-analysis was conducted to assess the improvement in Mini-Mental State Examination (MMSE) and Montreal Cognitive Assessment (MoCA) scores under a variety of non-pharmacological interventions.

**Results:**

A total of 22 RCTs involving 2,111 patients and 14 different non-pharmacological treatments were included. These interventions were transcranial direct current stimulation (tDCS), reminiscence therapy (RT), remote ischemic conditioning (RIC), physical fitness training (PFT), intensive patient care program (IPCP), moderate-intensity continuous training + high-intensity interval training (MICT + HIIT), medium intensity continuous training (MICT), grip training (GT), acupuncture, cognitive behavioral therapy (CBT), cognitive rehabilitation training (CRT), high pressure oxygen (HPO), moxibustion, and repetitive transcranial magnetic stimulation (rTMS). The results of the network meta-analysis indicated that rTMS had the highest likelihood of being the most effective intervention for improving MMSE and MoCA scores.

**Conclusion:**

The evidence from this study suggests that rTMS holds promise for improving MMSE and MoCA scores in patients with cognitive impairment following ischemic stroke. However, further high-quality research is needed to confirm and validate this finding.

## 1 Background

Ischemic stroke is a common clinical condition among middle-aged and elderly populations, with high incidence and disability rates in the world (1). However, the incidence of this condition has also been increasing among younger individuals in recent years (2). In a multi-country study, the incidence of cognitive impairment after stroke varied widely, ranging from 20 to 80% (3). Notably, in China, 78.7% of ischemic stroke patients were found to experience cognitive impairment (4), often within the first year after the onset of the disease (5). These impairments encompass various aspects of cognitive function, including attention, memory, executive function, language, and visual–spatial abilities. They significantly impact the daily lives of patients, hinder the rehabilitation for motor, speech, and swallowing functions, and impose substantial economic and psychological burdens on both patients and their families. Previous research has established that post-stroke cognitive impairment (PSCI) increases the risk of recurrent strokes, particularly ischemic strokes. For patients lacking reliable imaging evidence, PSCI can serve as an independent predictor of stroke recurrence (6). Unfortunately, there has been a disproportionate emphasis on the rehabilitation of motor and swallowing functions following ischemic stroke, with insufficient attention given to cognitive recovery (7). Currently, there are no specific drugs for cognitive impairment after ischemic stroke. Therefore, safe and effective treatment options are needed to manage cognitive impairment following cerebral infarction. In recent years, an increasing body of research has focused on non-pharmacological interventions to mitigate cognitive impairment after ischemic stroke. However, the comparative advantages of these treatments are still uncertain.

Network meta-analysis enables indirect comparisons and quantitative evaluation of different treatments to determine the most effective approach (8). Hence, this study employs network meta-analysis to investigate the impact of non-pharmacological interventions on improving cognitive impairment in patients with ischemic stroke, aiming to provide guidance for clinical practice.

## 2 Data and methods

This study has been registered on the PROSPERO platform with the registration number CRD42023456667. The PRISMA extension statement for reporting of systematic reviews incorporating network meta-analyses of healthcare interventions: checklist and explanations (8).

### 2.1 Literature screening

A combination of MeSh terms and free-text terms was applied to retrieve studies from Cochrane Library, PubMed, EmBase, and Web of Science databases until 15 March 2023. Given the insufficiency of included studies, additional searches were conducted in Chinese databases including CNKI, WanFang, and VIP until 5 May 2023. The specific search strategy is shown in Supplementary Table S1.

### 2.2 Inclusion and exclusion criteria

#### 2.2.1 Study type: randomized controlled trials

#### 2.2.2 Study population: patients with post-ischemic stroke cognitive impairment

#### 2.2.3 Intervention measures

Experimental group: Reminiscence therapy (RT), remote ischemic conditioning (RIC), physical fitness training (PFT), intensive patient care program (IPCP), grip training (GT), cognitive behavioral therapies (CBT), cognitive rehabilitation training (CRT), high-pressure oxygen therapy (HPO), repetitive trans-cranial magnetic stimulation (rTMS), trans-cranial direct current stimulation (tDCS), moxibustion, moderate intensity continuous training (MICT), and moderate-intensity continuous training + high-intensity interval training (MICT + HIIT). Control group: bank control group. The control group received standard clinical treatments such as anticoagulation, lipid regulation, and plaque stabilization but no cognition-improving medications. Both the control and experimental groups underwent the same rehabilitation training.

#### 2.2.4 Outcome measures

(1) Mini-Mental State Examination (MMSE). (2) Montreal Cognitive Assessment Scale (MoCA).

#### 2.2.5 Exclusion criteria

(1) Non-English or Non-Chinese literature. (2) Duplicate publications. (3) Lack of usable outcome measures. (4) Data errors or unobtainable data, even after attempting to contact the authors. (5) Experimental or control groups receiving drugs for improving cognitive impairment. These drugs have the effect of improving cognitive function on the drug instructions, and the purpose of drug research is to improve cognitive function.

### 2.3 Data extraction

Two reviewers independently screened literature, extracted data, and cross-verified the information. In the case of disagreements, resolution was achieved through discussion or consultation with a third reviewer. The literature was screened by first reading the titles, and after excluding obviously irrelevant articles, abstracts and full texts were further examined to determine eligible studies. If necessary, attempts were made to contact the original authors of the studies via email or phone to obtain critical information that was uncertain but vital for this study. Extracted data included (1) basic information of included studies: study title, first author, country, publication year, etc.; (2) baseline characteristics of study subjects and interventions; (3) key elements of risk of bias assessment; (4) outcome measures of interest and outcome measurements.

### 2.4 Quality assessment

Two researchers independently assessed the risk of bias of included randomized controlled trials (RCTs) using the bias risk assessment tool which was recommended in Cochrane Handbook version 5.1.0. The results were cross-verified by both reviewers, and in the case of discrepancies, resolution was determined through discussion between or consultation with a third reviewer. The assessment covered seven aspects, with options for each item being “low risk,” “high risk,” or “unclear risk.”

### 2.5 Statistical analysis

In this study, a Bayesian network meta-analysis was conducted using R version 4.2.3, RStudio software, and the GeMTC package. After conducting consistent and non-consistent modeling, the Deviance Information Criterion (DIC) for the MMSE is 60.38850, while the DIC for the MoCA is 72.22438. These results indicate that the model shows consistency in its performance (9). Fixed-effects models were employed to summarize the effect estimates from different studies. The combined effect size was described using the mean difference (MD) and a 95% confidence interval (CI). The results were presented through forest plots, league tables, and cumulative probability ranking plots. The surface under the cumulative ranking curve (SUCRA) was calculated to indicate the likelihood of each intervention being the best. SUCRA values range from 0 to 1, and interventions were ranked based on their SUCRA values. Network diagrams and comparison-adjusted funnel plots were created using Stata 15. Publication bias risk was visualized using Review Manager 5.4.1.

## 3 Results

### 3.1 Literature search flow and results

**Figure 1:**
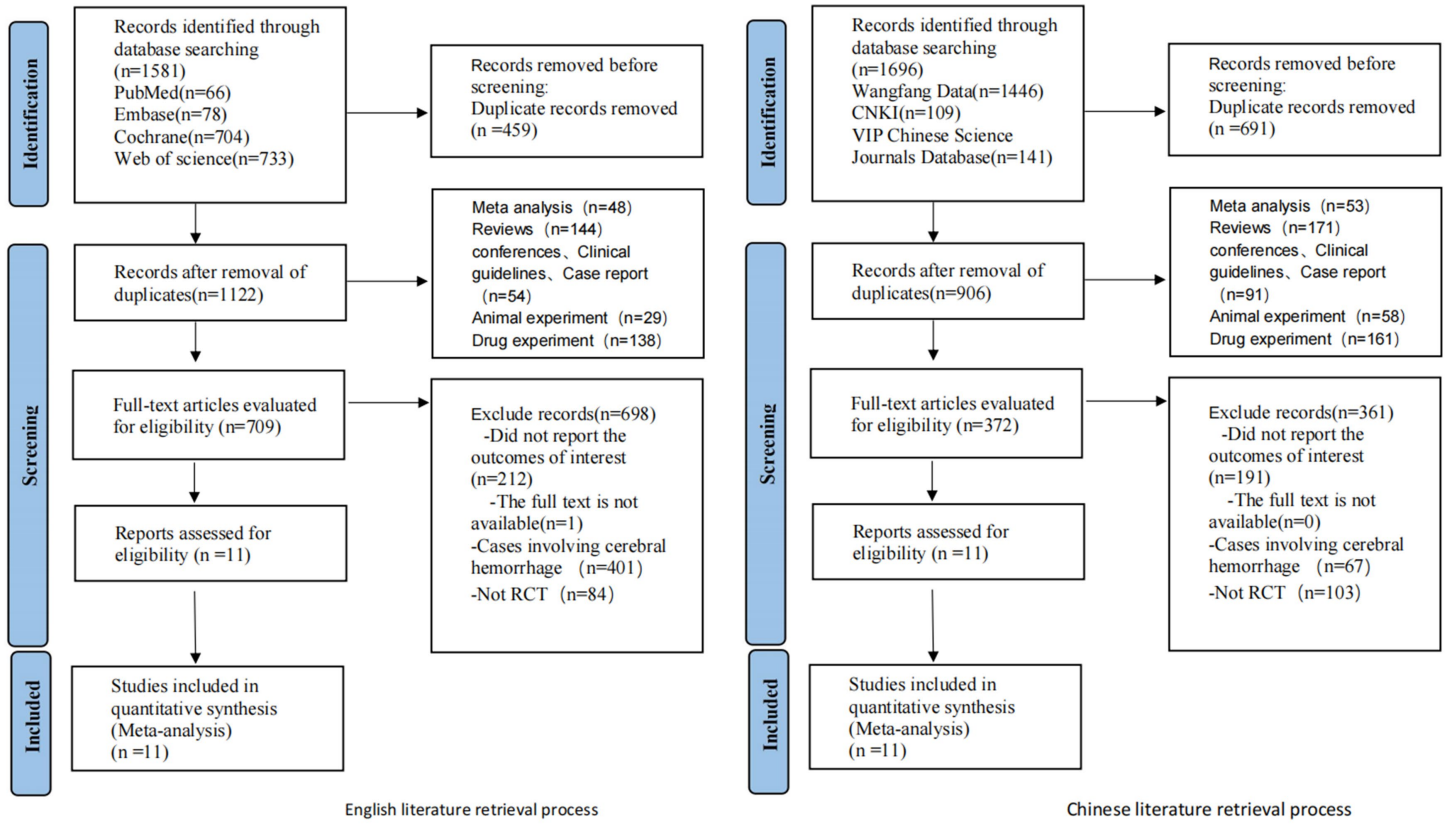
Literature search flow.

Initial screening yielded 1,581 relevant English articles. Following a layered selection process, 11 RCTs were ultimately included. Due to the limited availability of eligible English literature and treatment modalities, we extended our search to Chinese databases including CNKI, WanFang, and VIP, resulting in the inclusion of 11 Chinese articles that met the aforementioned inclusion and exclusion criteria, totaling 22 RCTs. A total of 22 RCTs involving 2,111 patients were included. Among these trials, 19 were from China, and one was from Brazil, one from the Netherlands, and one from Canada. Fourteen non-pharmacological interventions were used, namely, tDCS, RT, RIC, PFT, IPCP, MICT + HIIT, MICT, GT, acupuncture, CBT, CRT, HPO, moxibustion, and rTMS. They were all compared in pairs with the control group. The selection process and results are shown in Figure 1.

### 3.2 Baseline characteristics of included studies

The basic characteristics of included studies are shown in Table 1.

**Table 1:** Characteristics of included studies.

Study	Year	Country	Sample-size	Gender (M/F)	Mean age	Intervention	Outcome
Boasquevisque et al. (10)	2021	Brazil	tDCS: 15	12/18	tDCS: 61.8	tDCS	MoCA
			Sham: 15		Sham: 61.9		
Li et al. (11)	2021	China	RT: 108	136/80	RT: 65.0	RT	MMSE
			Sham: 108		Sham: 66.0		
Mi et al. (12)	2016	China	RIC: 9	45,117	RIPC: 67.0	RIC	MoCA
			Sham: 8		Sham: 59.5		MMSE
Zhou et al. (13)	2018	China	RIC: 30	35/23	RIC: 83.5	RIC	MoCA
			Sham: 28		Sham: 84.2		MMSE
Li et al. (14)	2020	China	RIC: 24	30/18	RIPC: 68.3	RIC	MoCA
			Sham: 24		Sham: 66.7		
Wang et al. (15)	2017	China	RIC: 14	15/15	RIC: 65.71	RIC	MoCA
			Sham: 16		Sham: 60.75		MMSE
Deijle et al. (16)	2022	Netherlands	PFT: 60	70/50	PFT: 64.7	PFT	MoCA
			Sham: 60		Sham: 63.9		
Yu et al. (17)	2019	China	IPCP: 121	149/93	IPCP: 67.3	IPCP	MMSE
			Sham: 121		Sham: 67.5		
Lapointe et al. (18)	2023	Canada	HIIT + MICT: 19	33/19	HIIT + MICT: 71.8	HIIT + MICT	MoCA
			MICT: 16		MICT: 65.6	MICT	
			Sham: 17		Sham: 69.2		
Shang et al. (19)	2021	China	GT: 37	41/35	GT: 63.68	GT	MoCA
			Sham: 39		Sham: 64.13		
Chen et al. (20)	2016	China	Acupuncture: 120	148/92	Acupuncture: 62.52	Acupuncture	MoCA
			Sham: 120		Sham: 64.06		MMSE
Huang et al. (21)	2008	China	Acupuncture: 40	41/39	Acupuncture: 59.22	Acupuncture	MMSE
			Sham: 40		Sham: 61.05		
Zhang et al. (22)	2020	China	Acupuncture: 30	38/22	Acupuncture: 70.10	Acupuncture	MoCA
			Sham: 30		Sham: 69.03		
Zhang et al. (23)	2011	China	CBT: 84	93/74	CBT: 84	CBT	MoCA
			Sham: 83		Sham: 83		
Zhang (24)	2013	China	CRT: 50	54/46	CRT: 65.1	CRT	MMSE
			Sham: 50		Sham: 63.5		
Su et al. (25)	2022	China	RT: 77	98/57	RT: 62.51	RT	MMSE
			Sham: 78		Sham: 63.47		
Duan et al. (26)	2018	China	HPO: 45	49/41	HPO: 58.3	HPO	MoCA
			Sham: 45		Sham: 55.8		
Yan et al. (27)	2018	China	Moxibustion: 45	53/47	Moxibustion: 65.65	Moxibustion	MMSE
			Sham: 45		Sham: 63.21		MoCA
Gao et al. (28)	2019	China	rTMS: 43	49/37	rTMS: 52.5	rTMS	MMSE
			Sham: 43		Sham: 52.3		
Zhu et al. (29)	2022	China	rTMS: 35	37/33	rTMS: 66.97	rTMS	MoCA
			Sham: 35		Sham: 66.84		MMSE
Li et al. (30)	2015	China	rTMS: 23	23/22	rTMS: 58.6	rTMS	MoCA
			Sham: 22		Sham: 57.9		
Liang et al. (31)	2018	China	RIC: 23	27//21	RIC: 67.91	RIC	MoCA
			Sham: 25		Sham: 66.52		MMSE

RT, reminiscence therapy; RIC, remote ischemic conditioning; PFT, physical fitness training; IPCP, intensive patient care program; GT, grip training; CBT, cognitive behaviour therapies; CTR, cognitive rehabilitation training; HPO, high pressure oxygen; rTMS, repetitive trans-cranial magnetic stimulation; tDCS, trans-cranial direct current stimulation.

### 3.3 Network relationships of interventions

**Figure 2:**
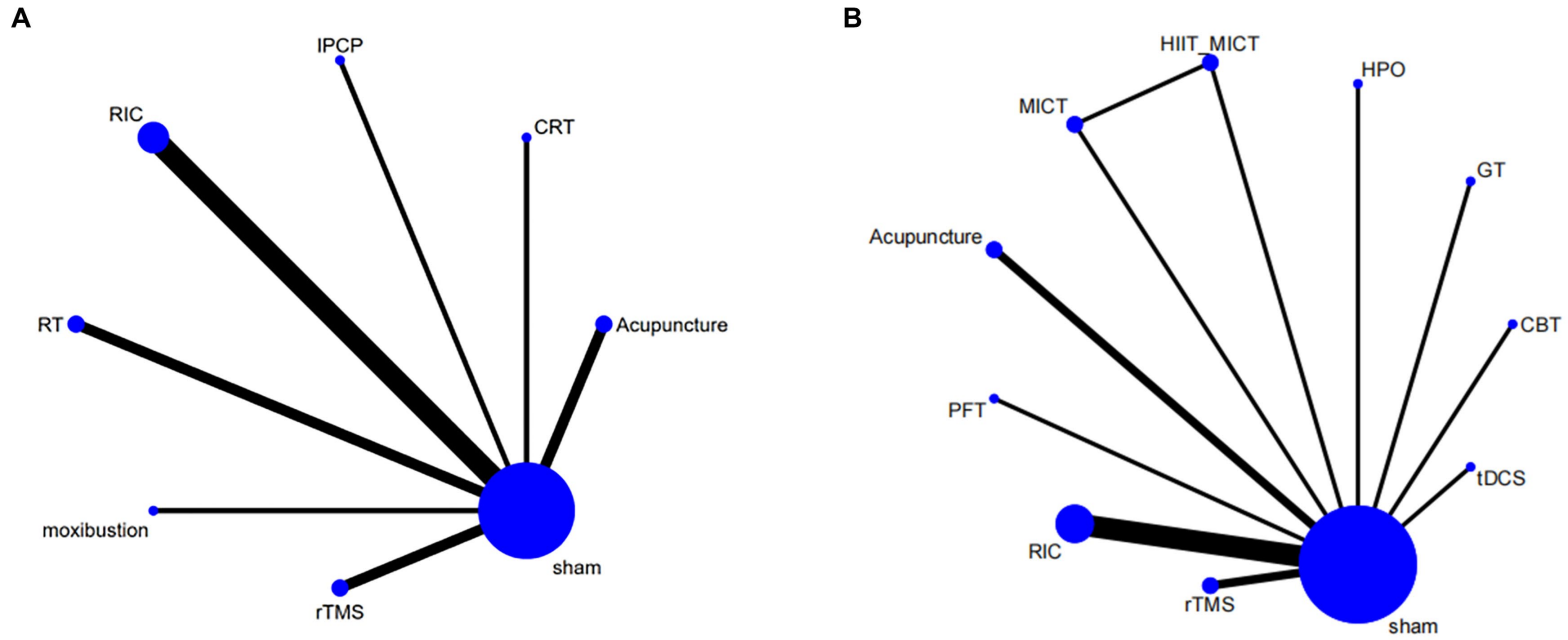
The network relationships of outcome measures **(A)** MMSE, **(B)** MoCA.

The intervention network diagram (Figure 2) displays all the available comparisons for the included trials. A direct relationship is indicated by a line between two circles, while no line indicates no direct relationship. The size of the circles represents the sample size of the interventions, and the thickness of the lines represents the number of studies included between the two interventions. In this study, 4 (12, 13, 15, 31), 2 (20, 21), 2 (28, 29), 2 (11, 25), 1 (23), 1 (17), and 1 (27) RCTs, respectively, compared RIC, acupuncture, rTMS, RT, CRT, IPCP, and moxibustion with the blank control group in terms of MMSE scores. Additionally, 5 (12–15, 31), 2 (20, 22), 2 (29, 30), 1 (23), 1 (19), 1 (26), 1 (16), 1 (27), and 1 (10) RCTs, respectively, compared RIC, acupuncture, rTMS, CBT, GT, HPO, PFT, moxibustion, and tDCS with the control group in terms of MoCA scores. One RCT compared HIIT + MICT with the control group in terms of MoCA scores.

### 3.4 Bias risk assessment chart

**Figure 3:**
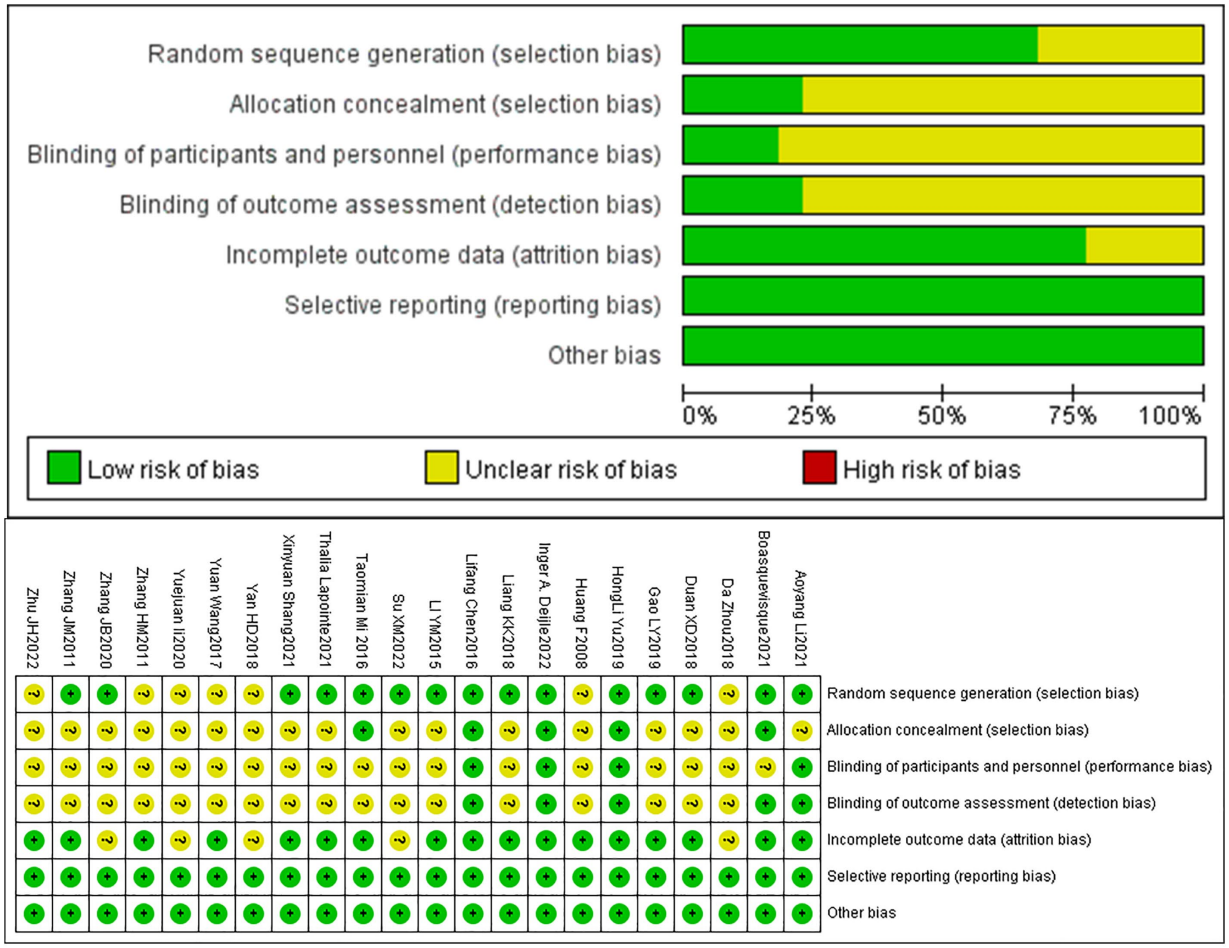
Bias risk assessment chart.

A total of 22 articles were included. Among them, 15 articles mentioned the use of randomization through techniques such as random number tables or envelope methods, while 7 articles simply mentioned the term “random.” Four articles provided detailed descriptions of the methods used for concealed allocation of sequences. Due to the nature of non-pharmacological treatments, achieving double-blinding of implementers and participants was challenging, Moreover, only 4 articles mentioned the implementation of double-blinding. Five articles mentioned blinding of outcome assessors. Four articles mentioned participant dropout and provided details about the groups from which participants dropped out and the specific reasons. All included articles had a relatively low probability of selective reporting bias and other sources of bias. The bias risk assessment results of included articles are presented in Figure 3.

### 3.5 Meta-analysis results

#### 3.5.1 MMSE scores

**Figure 4:**
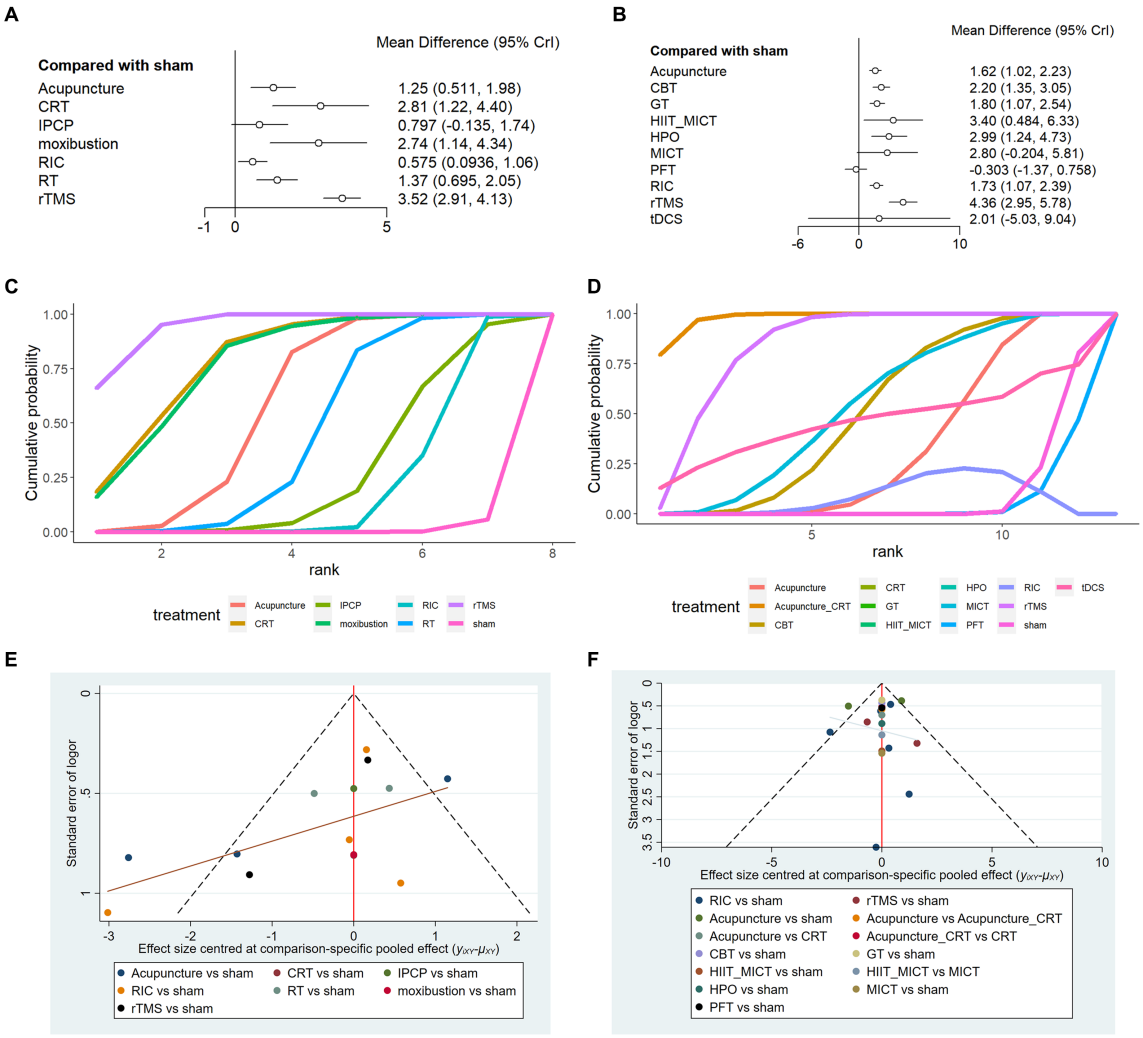
Randomized controlled trial **(A)** RCT of MMSE, **(B)** RCT of MoCA; **(C)** line chart of MMSE, **(D)** line chart of MoCA; **(E)** funnel plot of MMSE, and **(F)** funnel plot of MoCA.

A total of 13 RCTs reported MMSE scores. The network meta-analysis results demonstrate that the increase in MMSE scores for rTMS, CRT, moxibustion, acupuncture, RT, IPCP, and RIC is higher compared with the control group. Details are presented in Figures 4A,B and Table 2. The SUCRA scores for the treatments are: rTMS (0.94) > CRT (0.80) > moxibustion (0.79) > RT (0.50) > acupuncture (0.46) > IPCP (0.29) > RIC (0.20) > control group (0.01). The results point to rTMS as the most likely intervention to improve MMSE scores in patients with ischemic stroke-related cognitive impairment. Figures 4A,B provide the cumulative probability ranking.

**Table 2:** MD 95% CI (MMSE).

Acupuncture							
−1.57 (−3.31, 0.19)	CRT						
0.45 (−0.74, 1.64)	2.01 (0.16, 3.85)^*^	IPCP					
−1.5 (−3.26, 0.28)	0.07 (−2.19, 2.33)	−1.94 (−3.8, −0.09)^*^	Moxibustion				
0.67 (−0.21, 1.55)	2.23 (0.57, 3.9)^*^	0.22 (−0.82, 1.28)	2.17 (0.5, 3.84)^*^	RIC			
−0.13 (−1.13, 0.87)	1.44 (−0.29, 3.17)	−0.58 (−1.72, 0.58)	1.37 (−0.37, 3.1)	−0.8 (−1.64, 0.03)	RT		
−2.27 (−3.23, −1.32)^*^	−0.71 (−2.42, 0.99)	−2.72 (−3.84, −1.61)^*^	−0.78 (−2.5, 0.94)	−2.95 (−3.72, −2.17)^*^	−2.15 (−3.06, −1.24)^*^	rTMS	
1.25 (0.51, 1.98)^*^	2.81 (1.22, 4.4)^*^	0.8 (−0.13, 1.74)	2.74 (1.14, 4.34)^*^	0.58 (0.09, 1.06)^*^	1.37 (0.7, 2.05)^*^	3.52 (2.91, 4.13)^*^	Sham

#### 3.5.2 MoCA scores

A total of 16 RCTs reported MoCA scores. The network meta-analysis results indicate that for MoCA scores, rTMS, MICT + HIIT, MICT, HPO, CBT, tDCS, GT, RIC, and acupuncture exhibit higher increases in MoCA scores compared with the control group. However, PFT shows a lower rise in MoCA scores in comparison to the control group. Details are shown in Figures 4A,B and Table 3. The SUCRA scores for the treatments are rTMS (0.92) > MICT + HIIT (0.76) > HPO (0.72) > MICT (0.64) > CBT (0.57) > tDCS (0.50) > GT (0.44) > RIC (0.42) > acupuncture (0.38) > control group (0.10) > PFT (0.06). The results point to rTMS as the most likely successful approach for rising MoCA scores in those with ischemic stroke-related cognitive impairment. The cognitive function is better when the MMSE and MoCA scores are higher. Thus, a higher cumulative area under the curve (SUCRA) of the intervention indicates a better effect on improving cognitive impairment in ischemic stroke. The cumulative probability line chart (Figures 4C,D) shows the same SUCRA, as shown in Figures 4A,B.

**Table 3:** MD 95% CI (MoCA).

Acupuncture										
−0.58 (−1.62, 0.47)	CBT									
−0.18 (−1.12, 0.77)	0.4 (−0.72, 1.53)	GT								
−1.77 (−4.76, 1.23)	−1.2 (−4.25, 1.87)	−1.6 (−4.61, 1.44)	HIIT_MICT							
−1.38 (−3.21, 0.47)	−0.8 (−2.73, 1.14)	−1.2 (−3.08, 0.69)	0.39 (−3.02, 3.82)	HPO						
−1.17 (−4.25, 1.92)	−0.6 (−3.74, 2.56)	−1 (−4.1, 2.13)	0.6 (−1.64, 2.83)	0.21 (−3.3, 3.69)	MICT					
1.92 (0.7, 3.15)^*^	2.5 (1.13, 3.86)^*^	2.1 (0.82, 3.38)^*^	3.69 (0.57, 6.81)^*^	3.3 (1.26, 5.33)^*^	3.09 (−0.11, 6.29)	PFT				
−0.11 (−1.01, 0.79)	0.47 (−0.62, 1.54)	0.07 (−0.92, 1.05)	1.66 (−1.36, 4.66)	1.27 (−0.6, 3.12)	1.06 (−2.06, 4.16)	−2.03 (−3.29, −0.78)	RIC			
−2.74 (−4.26, −1.22)	−2.17 (−3.81, −0.53)	−2.57 (−4.14, −0.99)	−0.97 (−4.22, 2.27)	−1.37 (−3.6, 0.86)	−1.57 (−4.92, 1.74)	−4.67 (−6.41, −2.91)	−2.63 (−4.17, −1.09)	rTMS		
1.62 (1.02, 2.23)^*^	2.2 (1.34, 3.05)^*^	1.8 (1.06, 2.53)^*^	3.4 (0.45, 6.32)^*^	3 (1.26, 4.73)^*^	2.79 (−0.25, 5.81)	−0.3 (−1.36, 0.76)	1.73 (1.07, 2.4)^*^	4.36 (2.96, 5.76)^*^	Sham	
−0.38 (−7.5, 6.69)	0.19 (−6.95, 7.29)	−0.21 (−7.33, 6.89)	1.38 (−6.31, 9.01)	0.99 (−6.33, 8.25)	0.79 (−6.92, 8.47)	−2.31 (−9.47, 4.85)	−0.28 (−7.39, 6.82)	2.36 (−4.87, 9.57)	−2.01 (−9.09, 5.04)	tDCS

### 3.6 Publication bias assessment

The publication bias funnel plot (Figures 4E,F) indicates that the funnel plots of the two outcome indicators are not entirely symmetric. In this study, some points fall outside the funnel plot, particularly at the bottom, which suggests the presence of a small sample effect and publication bias. Therefore, it is important to interpret the research findings with caution.

## 4 Discussion

Currently, there is a significant clinical interest in the prevention and treatment of ischemic stroke post-cognitive impairment. Severe cognitive impairment has a profound effect on a patient’s recovery, covering motor skills, speech, and swallowing, in addition to preventing them from recovering their everyday life competence, hence intensifying the responsibility of family caretakers. Addressing cognitive impairment is therefore a priority for ischemic stroke patients to recover. Currently, there is much controversy over non-drug treatments for cognitive impairment after ischemic stroke, with no standardized protocols in place. Numerous investigations do not make a distinction between ischemic and hemorrhagic strokes, hence limiting their applicability to medical practice. The purpose of this research is to comprehensively examine the effectiveness of numerous non-pharmacological interventions in combating cognitive impairment due to ischemic stroke.

During the literature screening process, it was observed that MMSE and MoCA are commonly utilized for assessing cognitive function post-ischemic stroke. While MoCA demonstrates higher sensitivity in detecting early cognitive impairment compared with MMSE (32), incorporating both tools as outcome indicators can enhance the reliability of research findings. According to the network meta-analysis, acupuncture, IPCP, RIC, rTMS, CRT, RT, and moxibustion are all likely to improve MMSE scores in a meaningful way. Of these, rTMS appears to be the most effective. Regarding MoCA scores, rTMS, HPOT, MICT, MICT + HIIT, CBT, TDCS, GT, RIC, and acupuncture all have a positive effect on cognitive function improvement. Nonetheless, PFT does not show a significant effect on cognitive enhancement, whereas rTMS is the most efficient approach for increasing MoCA scores.

Cumulative results from both measures imply that rTMS is the optimal approach for enhancing cognitive abilities following ischemic stroke. However, the specific mechanisms remain unclear. Previous research suggests that rTMS can improve cerebral blood flow (33, 34), enhance brain metabolism (35–37), and repair nerve function (38). Hong et al. (38) has found that 10 Hz of rTMS inhibits the neurotoxic transformation of astrocytes following focal cerebral ischemia.

Deijle et al. (16) and Lapointe et al. (18) have found that simple PFT has no impact on cognitive function in ischemic stroke patients. Cognitive function improvement is only observed when combining cognitive training with procedural guidance in addition to physical exercise (39, 40). The study by Shang et al. (19) on GT indicates that it may not only improve motor function but also potentially enhance cognitive function. This suggests that grip actions may induce changes in certain brain regions, and white matter remodeling might be a contributing factor in the cognitive improvement which is observed after early-stage, strength-enhancing grip training following a stroke. The findings by Boasquevisque et al. (10) attest to the fact that tDCS of the ipsilesional dorsolateral prefrontal cortex does not result in enhanced cognitive performance in subjects affected by ischemic stroke. Research by André et al. (41) demonstrates that applying anode tDCS to the left dorsolateral prefrontal cortex can enhance visual short-term memory in individuals with mild vascular dementia. Previous research has suggested that tDCS may improve daily activities, yet its effects on upper and lower limb capability, power, and cognitive performance remain undetermined. Further study is required on parameters, such as stimulation type, stimulation location, duration, intensity, and electrode size and positioning (42).

Studies by Zhou et al. (13), Liang et al. (31), Mi et al. (12), Wang et al. (15), and Li et al. (14) point out the beneficial effects of RIC on cognitive recovery after ischemic stroke. The chosen parameters were largely consistent, including a 5 min cycle of compression and release. Compression can be administered bilaterally or to the non-paralyzed side with pressure of 20–30 mmHg greater than systolic pressure or a maximum of 200 mmHg. Our research results show the considerable effect of RIC on the cognitive progress of ischemic stroke patients. Evidence gathered by Zhao et al. (43) indicates that RIC displays a neuroprotective effect, as it effectively reduces the rate of recurrence of ischemic stroke and transient cerebral ischemia. Furthermore, research by Liang et al. (31) suggests that RIC may exert a protective effect on cognitive impairment following ischemic stroke by upregulating the expression of HSP27 and HSP70.

Results of this study suggest that acupuncture can aid in the restoration of cognitive function following an ischemic stroke. Investigations concerning the use of acupuncture in ischemic stroke recovery have mainly centered on both motor functions and swallowing abilities while paying less attention to the cognitive functions and much less to the particular mechanisms and acupuncture points. Currently, it is generally accepted that when addressing motor disability in acute ischemic stroke patients, one should also take into account swallowing, cognitive, and other functional impairments. In an experiment by Zhang et al. (22), MS1 and MS6 were the acupoints of choice, whereas investigation by Chen et al. (20) did not designate any specific points but emphasized remedying all functional impairments via acupuncture. The study conducted by Yan et al. (27) examined the influences of moxibustion on the Dazhui and Shenshu acupoints, providing evidence of positive impacts on cerebral functioning. Studies suggest that acupuncture may promote the regeneration of functionally replaced cells and the recovery of damaged and newly generated neurons (44).

According to the research by Li et al. (11) and Su et al. (25), RT has been shown to be efficacious in improving cognitive capacities in acute ischemic stroke patients, in addition to alleviating symptoms of depression and anxiety, thus leading to an improved prognosis for the patients. Previous studies have confirmed the positive effects of RT on dementia patients in terms of quality of life, cognition, communication, and emotions. Through RT, it may be possible to evoke meaningful or joyful past occurrences, thereby stimulating thinking and slowing down memory decline. Moreover, RT can alleviate anxiety and unease, bolster a desire for a more promising future, and encourage adherence to rehabilitation protocols and treatment, ultimately improving patient prognosis (45).

In our daily clinical practice, CRT is widely used to strengthen cognitive function without distinguishing between ischemic stroke and cerebral hemorrhage patients. According to the investigation conducted by Zhang (24), it is essential to individualize treatment regimens based on specific cognitive levels to enhance cognitive capacity in patients. The research by Zhang et al. (23) has demonstrated that CBT may increase cognitive capabilities by means of shaping, punishment, rewards, and modeling. Furthermore, the study by Yu et al. (17) highlights the significance of creating a supportive social environment for individuals with cognitive impairments through long-term communication follow-ups, CRT, and psychological counseling. Studies have indicated that when patients with post-stroke cognitive impairment are exposed to an enriching atmosphere, they tend to be more willing to take part in cognitive training and express their feelings, which can help with the rehabilitation of their cognitive abilities (46). The study by Duan et al. (26) has shown that through HPO, a notable decrease in P300 latency and a rise in amplitude have been observed, demonstrating an enhancement of cognitive function in ischemic stroke patients. As demonstrated by the research by Marcinkowska et al. (47), there is a lack of important evidence to suggest that HPO can bolster cognitive function. To accurately measure the effects of HPO on neuropsychologic deficits, more accurate neuropsychological assessment techniques are needed.

The current study is limited by the small number of studies and high-quality RCTs. Moreover, the majority of studies are considered cognitive function as one of the observed outcomes, and some RCTs did not prioritize the improvement in cognitive function initially. Additionally, this study focuses on the impact of specific therapies on cognitive function and does not explore the combined use of therapies. We hope for more attention and RCT studies on patients with cognitive impairments after ischemic stroke, as the restoration of cognitive function is crucial for rehabilitation in terms of motor, speech, and swallowing functions.

## 5 Conclusion

The evidence of this study suggests that rTMS is the most successful approach for advancing cognitive abilities in ischemic stroke patients. In addition, RIC, acupuncture, moxibustion, CBT, CRT, RT, and GT have a positive effect on the restoration of cognitive function in these patients. Moreover, a favorable social environment is also essential. The effectiveness of HPO is debatable in this population. Physical exercise alone does not lead to cognitive function improvement. These discoveries can be utilized to direct the selection of different non-pharmacological interventions in the clinical practice.

## Data availability statement

The original contributions presented in the study are included in the article/Supplementary material, further inquiries can be directed to the corresponding author.

## Author contributions

GY: Writing – original draft, Methodology, Formal analysis, Conceptualization. LG: Writing – review & editing, Supervision, Resources, Conceptualization. YZ: Writing – review & editing, Methodology, Investigation, Formal analysis. SL: Writing – review & editing, Investigation, Formal analysis, Data curation.
