# Supplementary material for: Network meta-analysis of non-pharmacological interventions for cognitive impairment after an ischemic stroke
Source: Front Neurol. 2024 Jun 4;15:1327065. doi: 10.3389/fneur.2024.1327065 (PMC11185141; doi:10.3389/fneur.2024.1327065)
Supplement: Supplementary file 1 [file Table_1.DOCX]

**Table S1 Search strategy for Pubmed**

| #1 | Ischemic Strokes [MeSH Terms] |
| --- | --- |
| #2 | (Ischemic Strokes[Title/Abstract]) OR (Stroke, Ischemic[Title/Abstract]) OR (Ischaemic Stroke[Title/Abstract]) OR (Ischaemic Strokes[Title/Abstract]) OR (Stroke, Ischaemic[Title/Abstract]) OR (Cryptogenic Ischemic Stroke[Title/Abstract]) OR (Cryptogenic Ischemic Strokes[Title/Abstract]) OR (Ischemic Stroke, Cryptogenic[Title/Abstract]) OR (Stroke, Cryptogenic Ischemic[Title/Abstract]) OR (Cryptogenic Stroke[Title/Abstract]) OR (Cryptogenic Strokes[Title/Abstract]) OR (Stroke, Cryptogenic[Title/Abstract]) OR (Cryptogenic Embolism Stroke[Title/Abstract]) OR (Cryptogenic Embolism Strokes[Title/Abstract]) OR (Embolism Stroke, Cryptogenic[Title/Abstract]) OR (Stroke, Cryptogenic Embolism[Title/Abstract]) OR (Wake-up Stroke[Title/Abstract]) OR (Stroke, Wake-up[Title/Abstract]) OR (Wake up Stroke[Title/Abstract]) OR (Wake-up Strokes[Title/Abstract]) OR (Acute Ischemic Stroke[Title/Abstract]) OR (Acute Ischemic Strokes[Title/Abstract]) OR (Ischemic Stroke, Acute[Title/Abstract]) OR (Stroke, Acute Ischemic[Title/Abstract]) |
| #3 | Cognitive Dysfunction [MeSH Terms] |
| #4 | (((((((((((((((((((((((((Cognitive Dysfunction[Title/Abstract]) OR (Cognitive Dysfunctions[Title/Abstract])) OR (Dysfunction, Cognitive[Title/Abstract])) OR (Dysfunctions, Cognitive[Title/Abstract])) OR (Cognitive Impairments[Title/Abstract])) OR (Cognitive Impairment[Title/Abstract])) OR (Impairment, Cognitive[Title/Abstract])) OR (Impairments, Cognitive[Title/Abstract])) OR (Cognitive Disorder[Title/Abstract])) OR (Cognitive Disorders[Title/Abstract])) OR (Disorder, Cognitive[Title/Abstract])) OR (Disorders, Cognitive[Title/Abstract])) OR (Mild Cognitive Impairment[Title/Abstract])) OR (Cognitive Impairment, Mild[Title/Abstract])) OR (Cognitive Impairments, Mild[Title/Abstract])) OR (Impairment, Mild Cognitive[Title/Abstract])) OR (Impairments, Mild Cognitive[Title/Abstract])) OR (Mild Cognitive Impairments[Title/Abstract])) OR (Cognitive Decline[Title/Abstract])) OR (Cognitive Declines[Title/Abstract])) OR (Decline, Cognitive[Title/Abstract])) OR (Declines, Cognitive[Title/Abstract])) OR (Mental Deterioration[Title/Abstract])) OR (Deterioration, Mental[Title/Abstract])) OR (Deteriorations, Mental[Title/Abstract])) OR (Mental Deteriorations[Title/Abstract]) |
| #5 | randomized controlled trial [Publication Type] OR randomized [Title/Abstract] OR placebo [Title/Abstract] |
| #6 | (#1 OR #2) AND (#3 OR #4) AND #5 |
